# Effects of miR-143 and its target receptor 5-HT2B on agonistic behavior in the Chinese mitten crab (*Eriocheir sinensis*)

**DOI:** 10.1038/s41598-021-83984-6

**Published:** 2021-02-24

**Authors:** Yang-Yang Pang, Gen-Yong Huang, Ya-Meng Song, Xiao- Zhe Song, Jia-Huan Lv, Long He, Chao Niu, Ao-Ya Shi, Xing-Liang Shi, Yong-Xu Cheng, Xiao-Zhen Yang

**Affiliations:** National Demonstration Center for Experimental Fisheries Science Education; Key Laboratory of Freshwater Aquatic Genetic Resources, Ministry of Agriculture; Engineering Research Center of Aquaculture, Shanghai Ocean University, No. 999, Huchenghuan Road, Shanghai, 201306 People’s Republic of China

**Keywords:** Animal behaviour, RNAi

## Abstract

Chinese mitten crab (*Eriocheir sinensis*) as a commercially important species is widely cultured in China. However, *E. sinensis* is prone to agonistic behavior, which causes physical damage and wastes energy resources, negatively impacting their growth and survival. Therefore, understanding the regulatory mechanisms that underlie the switching of such behavior is essential for ensuring the efficient and cost-effective aquaculture of *E. sinensis*. The 5-HT2B receptor is a key downstream target of serotonin (5-HT), which is involved in regulating animal behavior. In this study, the full-length sequence of 5-HT2B gene was cloned. The total length of the 5-HT2B gene was found to be 3127 bp with a 236 bp 5′-UTR (untranslated region), a 779 bp 3′-UTR, and a 2112 bp open reading frame encoding 703 amino acids. Phylogenetic tree analysis revealed that the 5-HT2B amino acid sequence of *E. sinensis* is highly conserved with that of *Cancer borealis*. Using in vitro co-culture and luciferase assays, the miR-143 targets the 5-HT2B 3′-UTR and inhibits 5-HT2B expression was confirmed. Furthermore, RT-qPCR and Western blotting analyses revealed that the miR-143 mimic significantly inhibits 5-HT2B mRNA and protein expression. However, injection of miR-143 did not decrease agonistic behavior, indicating that 5-HT2B is not involved in the regulation of such behavior in *E. sinensis*.

## Introduction

The Chinese mitten crab (*Eriocheir sinensis*) is widely cultured in China as a highly popular and nutritious food source. Accordingly, it is often managed at high stocking densities. However, competition for food and shelter under such conditions can lead to agonistic behavior^1^, which negatively impacts crab integrity, survival, and growth, ultimately resulting in economic loss. Thus, a deeper understanding of the mechanisms underlying agonistic behavior in *E. sinensis* is required to decrease the occurrence of such behavior and ensure efficient and cost-effective aquaculture.

Agonistic behavior is influenced by both intrinsic (e.g. sex, body, and chela size variations and reproductive state), and extrinsic (e.g. shelter, food, mating opportunity and mating territory) factors. From a physiological point of view, 5-HT is an important neurotransmitter that has been shown to regulate agonistic behavior in crustaceans such as *Homarus americanus*, *Procambarus clarkii*, *Orconectes rusticus*, and *Litopenaeus vannamei*^2–8^. Furthermore, our own research has demonstrated that injecting *E. sinensis* with 5-HT affects its agonistic behavior^9^. However, the mechanisms by which such effects manifest are not fully understood, and it is thought that 5-HT regulates agonistic behavior through different pathways. For instance, in a study on *Drosophila*, Alekseyenko et al. (2019) found that 5-HT modulates aggressive behavior through the GABAergic and cholinergic systems^10^. Furthermore, two separate studies have demonstrated that crustacean hyperglycemic hormone (CHH) levels are correlated with aggression in crustaceans^9,11^, and 5-HT is known to regulate the release of CHH^11,12^.

5-HT mediates agonistic behavior through its interactions with several receptor subtypes in animals. For example, the 5-HT1A receptor is involved in modulating aggression in *Drosophila*^10^, and injecting *Drosophila melanogaster* with the 5-HT2 receptor antagonist (R)-1-[2,5-dimethoxy-4-iodopheny l]-2-aminopropane decreases its overall aggression^13^. Our own studies have shown that 5-HT1B, 5-HT2B, and 5-HT7 mRNA levels in *E. sinensis* were significantly changed after fighting^9^. Furthermore, we injected *E. sinensis* with ketanserin tartrate (ketanserin) to research the function of the 5-HT2B receptor in the regulation of agonistic behavior. The result showed that injection of ketanserin can inhibit the agonistic behavior but does not decrease the 5-HT2B mRNA level^14^.

Ketanserin is a specific inhibitor of the 5-HT2 receptor. However, there are two 5-HT2 receptor subtypes (5-HT2A and 5-HT2B) in crustaceans. Ketanserin may inhibit the 5-HT2A receptor expression rather than 5-HT2B in *E. sinensis.* Accordingly, we speculated that the two 5-HT2 receptor subtypes play different roles in the regulation of agonistic behavior in *E. sinensis*. Furthermore, Majeed et al. (2014) had previously demonstrated that the dysfunction of 5-HT2B receptor during development negatively influences locomotive behavior in *D. melanogaster*^15^. Clearly, 5-HT2B and its relationship with agonistic behavior in *E. sinensis* is worthy of further study.

MicroRNAs (miRNAs) constitute a class of non-coding RNAs with lengths ranging from 18 to 22 nt that play important roles in many biological processes^16,17^. They are known to mediate neurogenesis in both vertebrates and invertebrates^18^ and act on the nervous system to regulate animal behavior^19^. For instance, Yin et al. (2019) reported that microRNA-143 (miR-143) affects pain-related behavior in mice through its expression in nociceptive neurons, and microRNA-200b (miR-200b) has been demonstrated to regulate attraction/aversion behavior via the dopaminergic and GABAergic systems in amphibians^20^. Studies have also shown that miR-96, which inhibits the 5-HT1B receptor, affects aggressive behavior in mice^21^. However, most studies on the behavioral effects of miRNAs have been performed using vertebrates or model organisms and not crustaceans.

Based on our knowledge of the importance of miRNAs in mediating animal behavior, we have previously hypothesized that miRNAs may target behavior-related genes and regulate agonistic behavior in *E. sinensis*. Accordingly, we have previously used a deep sequencing approach to explore the miRNA profile of *E. sinensis* during agonistic behavior^22^ and investigated the effects of serotonin (5-HT) in its regulation^9,14^. From the miRNA profile of *E. sinensis*, combined with the two computational algorithms miRanda and TargetScan 5.1, miR-143, miR-200b, and miR-429 were predicted to target 5-HT2B 3′UTR.

Accordingly, in the present study, we have cloned the full-length sequence of the 5-HT2B gene in *E. sinensis* for the first time. Furthermore, its miR-143, which targets 5-HT2B, was characterized using several methods. Our findings demonstrate that miR-143 inhibits the expression of 5-HT2B in *E. sinensis* by targeting the 5-HT2B 3′-UTR but does not affect its agonistic behavior. This result indicates that 5-HT2B can’t inhibit the agonistic behavior in *E. sinensis*.

## Results

### 5-HT2B gene and homology analysis

**Figure 1:**
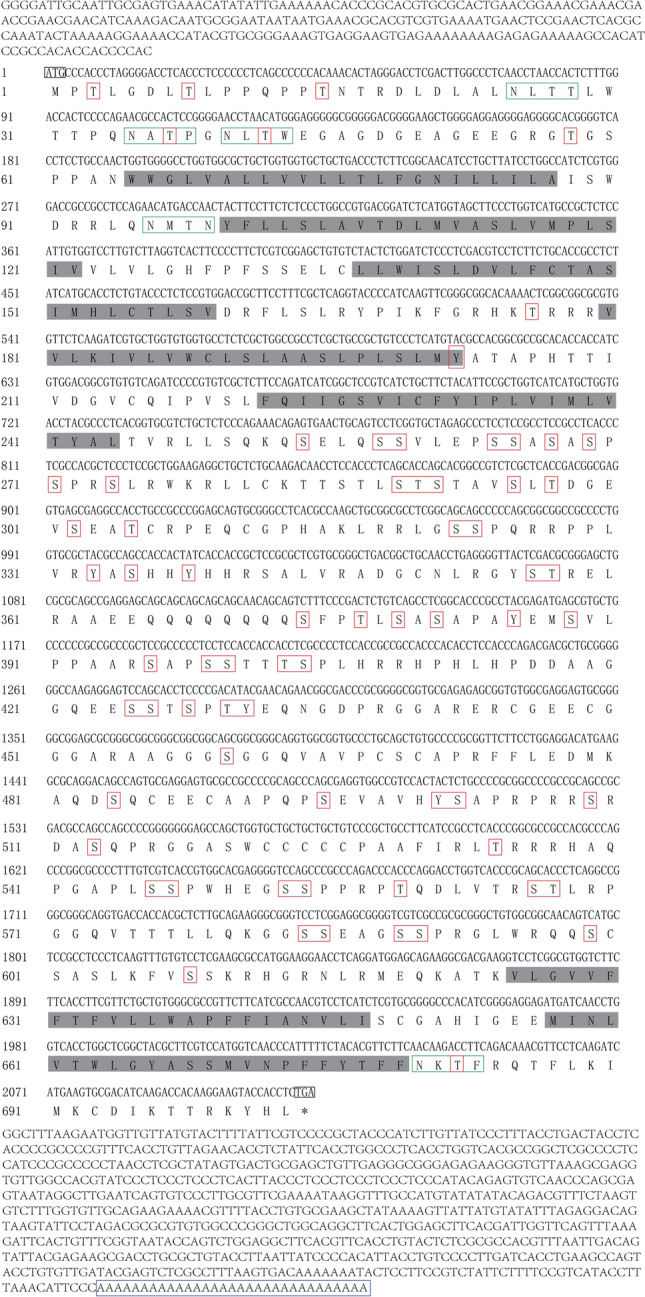
5-HT2B receptor cDNA sequence for *E. sinensis* and its predicted amino acid sequence. The coding area was enumerated from the 236 bp end, and the base sequence is shown under the corresponding amino acid sequence. The start codon (ATG) and the stop codon (TGA) are highlighted by black lines. The red boxes delineate the putative phosphorylation sites, and the putative N-glycosylation sites are shown in green boxes. The shaded area shows the seven transmembrane regions. The blue box indicates the poly (A) tail. 5-HT2B receptor cDNA sequence for *E. sinensis* has been uploaded to NCBI, and the accession number of 5-HT2B is MT670351.

The full-length 5-HT2B cDNA is 3127 bp and contains 236 bp 5′-UTR and a 779 bp 3′-UTR. In addition, the ORF has a sequence length of 2112 bp (including termination codon) and encodes 703 amino acids (Fig. 1). The 5-HT2B ORF sequence has been uploaded to GenBank and assigned the sequence ID MT670351. The results of the online analysis revealed that there are seven transmembrane domains in the 5-HT2B amino acid, but there is no signal peptide sequence for the N-terminal (Fig. 1). In addition, the N-glycosylation sites (N-x-[S/T]) and phosphorylation sites (S/T-x-[R/k]) are relatively conservative (Fig. 1).

**Figure 2:**
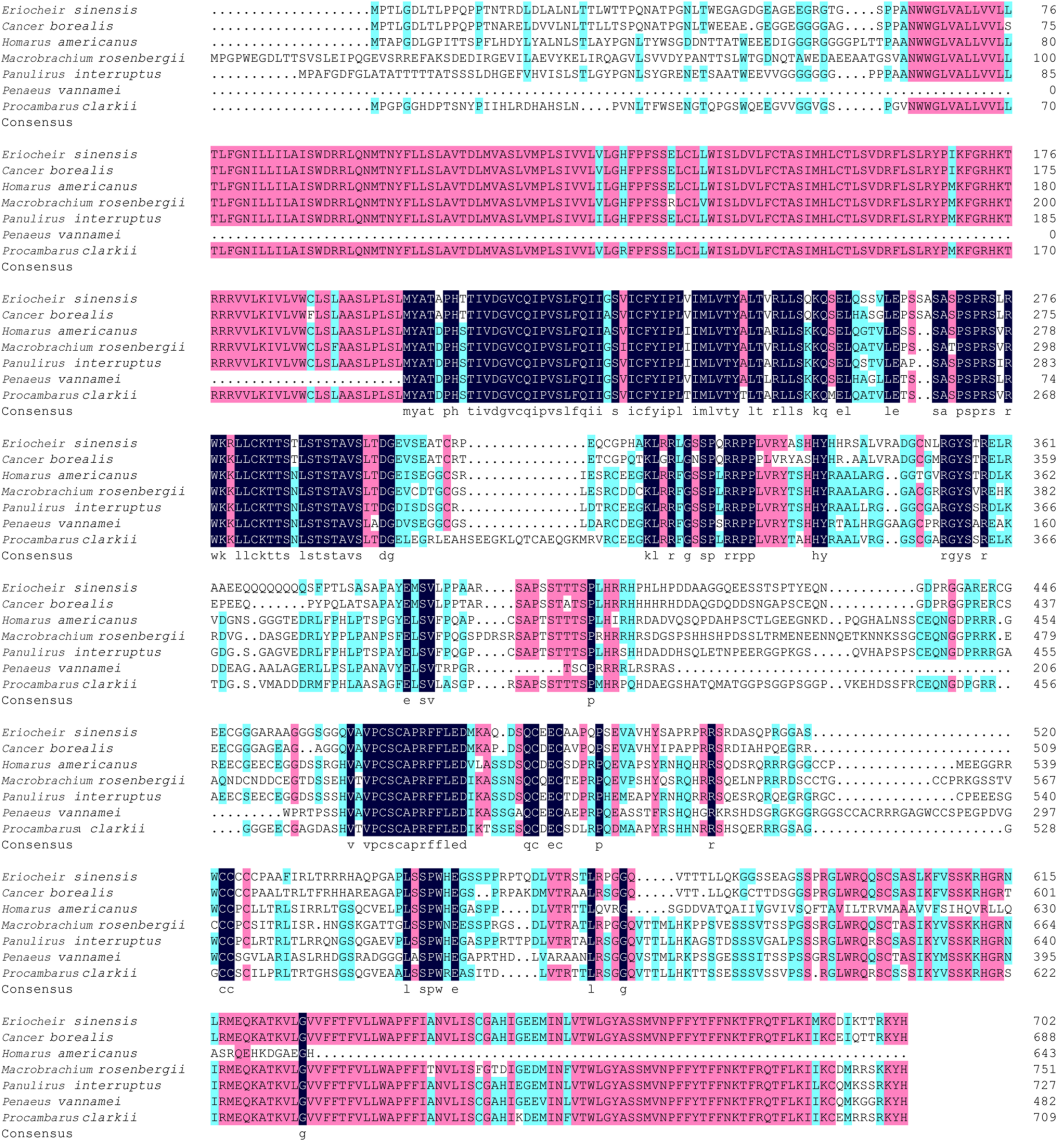
Alignment of the *E. sinensis* 5-HT2B gene amino acid sequence with those of other crustaceans. Conserved residues are highlighted in black. The 5-HT2B gene sequences of *C. borealis* (AOG14376.1), *H. americanus* (AOG12997.1), *M. rosenbergii* (ABM01873.1), *P. interruptus* (AAS57919.1), *P. vannamei* (ROT60950.1), and *P. clarkii* (ABX10972.1) were obtained from the NCBI.

**Figure 3:**
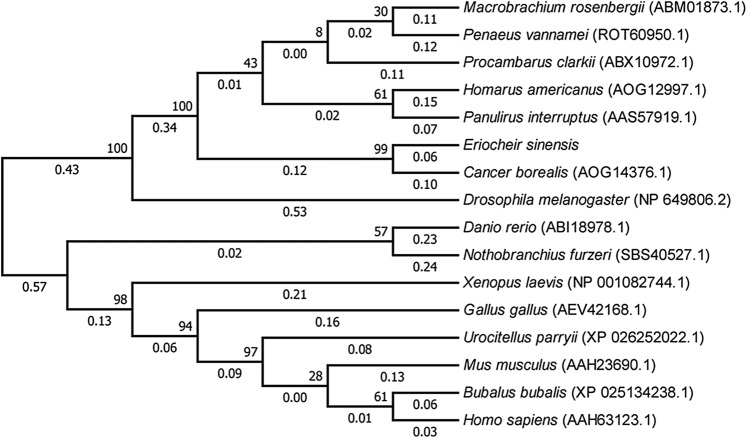
Phylogenetic relationship of the 5-HT2B gene between *E. sinensis* and other invertebrates and vertebrates. Bootstrap values from 1000 replicates are indicated at the nodes.

Multiple sequence alignment revealed that the 5-HT2B amino acid sequence of *E. sinensis* is highly conserved with those of *C. borealis*, *H. americanus*, *M. rosenbergii*, *P. interruptus*, and *P. clarkii* (Fig. 2). MEGA 7.0 software was used to construct the phylogenetic tree for the 5-HT2B gene of *E. sinensis* and several invertebrates and vertebrates. It was found that the 5-HT2B genes of *E. sinensis* and *C. borealis* are clustered into one branch, indicating that these two species are closely related. Furthermore, there is a certain genetic relationship between *E. sinensis* and *Drosophila* (Fig. 3). 5-HT2B of *E. sinensis* is located on a different branch to those of aquatic vertebrates (zebrafish), amphibians (*Xenopus laevis*), and mammals (mice) (Fig. 3). This result is consistent with the development of molecular homology and biological evolution.

### 5-HT2B expression in tissues co-cultured with miRNA mimics

**Figure 4:**
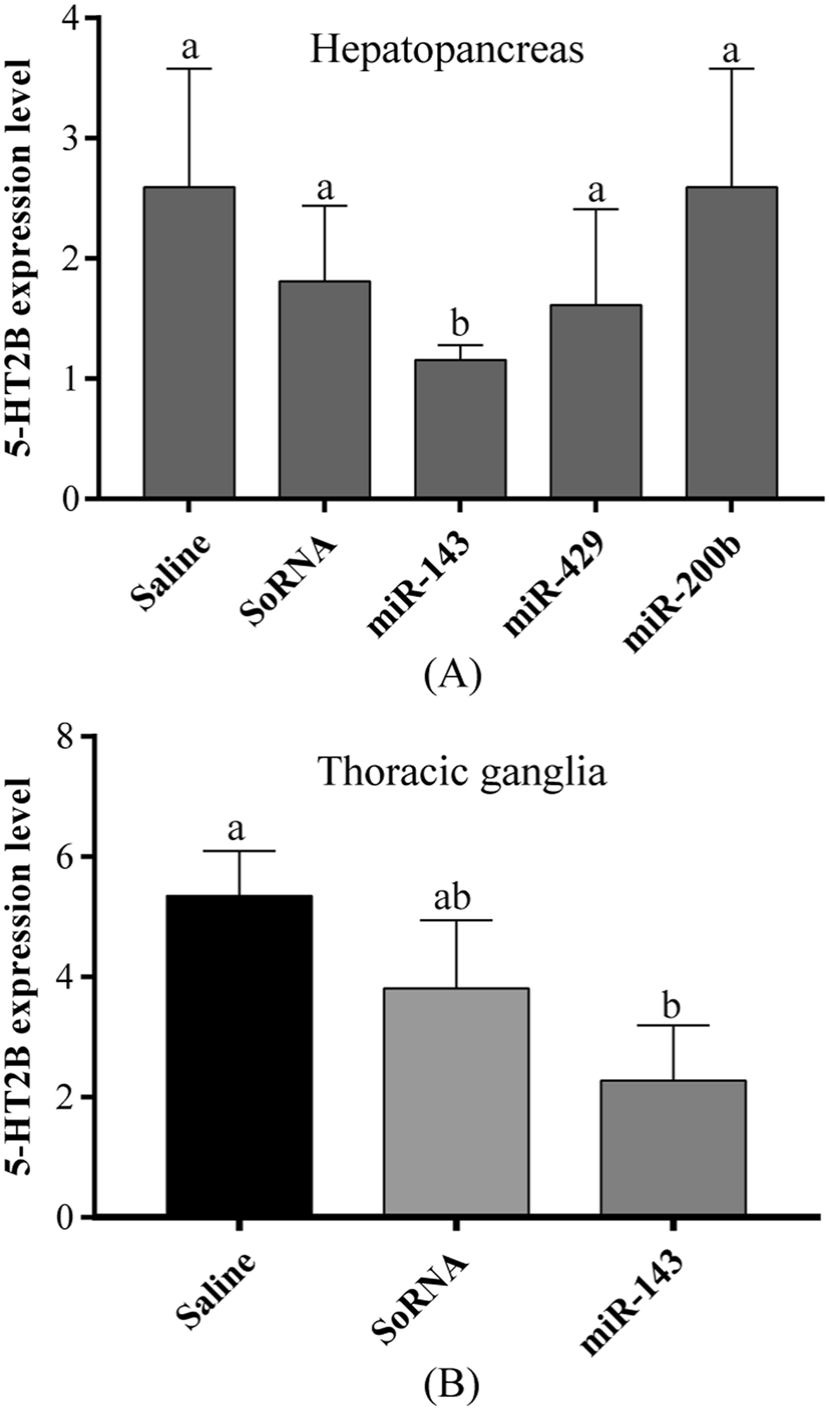
(**A**) Co-culture of the hepatopancreas with different miRNA mimics affects the expression level of the 5-HT2B receptor; (**B**) co-culture of the thoracic ganglion with an miR-143 mimic affects the expression level of the 5-HT2B receptor. Different letters above the horizontal line indicate significant differences between two groups (*P* < 0.05).

The miR-143 mimic, miR-429 mimic, and miR-200b mimic were co-cultured with the hepatopancreas of *E. sinensis*. We found that miR-143 significantly inhibits the expression of 5-HT2B mRNA (*P* < 0.05), but miR-429 and miR-200b does not (Fig. 4A). In order to confirm that miR-143 could decrease 5-HT2B expression in the nerve tissue of *E. sinensis*, we then co-cultured miR-143 mimic with thoracic ganglion tissue, and the results confirmed that miR-143 significantly inhibits 5-HT2B mRNA expression (*P* < 0.05, Fig. 4B).

### miR-143 targets the 5-HT2B 3′UTR

**Figure 5:**
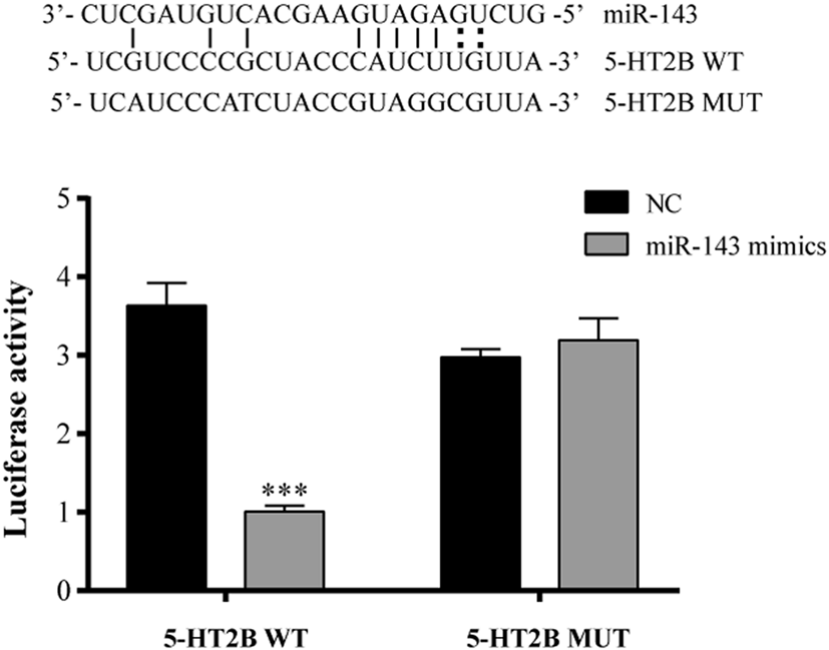
Relative luciferase activity for different groups co-transfected with an miR-143 mimic (5-HT2B-WT or 5-HT2B-MUT vectors). The asterisks above the horizontal line indicate significant differences between 5-HT2B-WT + miR-143 mimic and 5-HT2B-WT + NC mimic groups (*P* < 0.05).

As shown in Fig. 5, co-transfection of the 5-HT2B-WT vector and miR-143 mimic significantly inhibits the relative activity of luciferase when compared with that of the NC group (*P* < 0.05). However, the miR-143 mimic does not affect the luciferase activity of the 5-HT2B-MUT vector. These results indicated that miR-143 effectively targets the 5-HT2B 3′UTR.

### Injection of the miR-143 mimic decreases 5-HT2B expression

**Figure 6:**
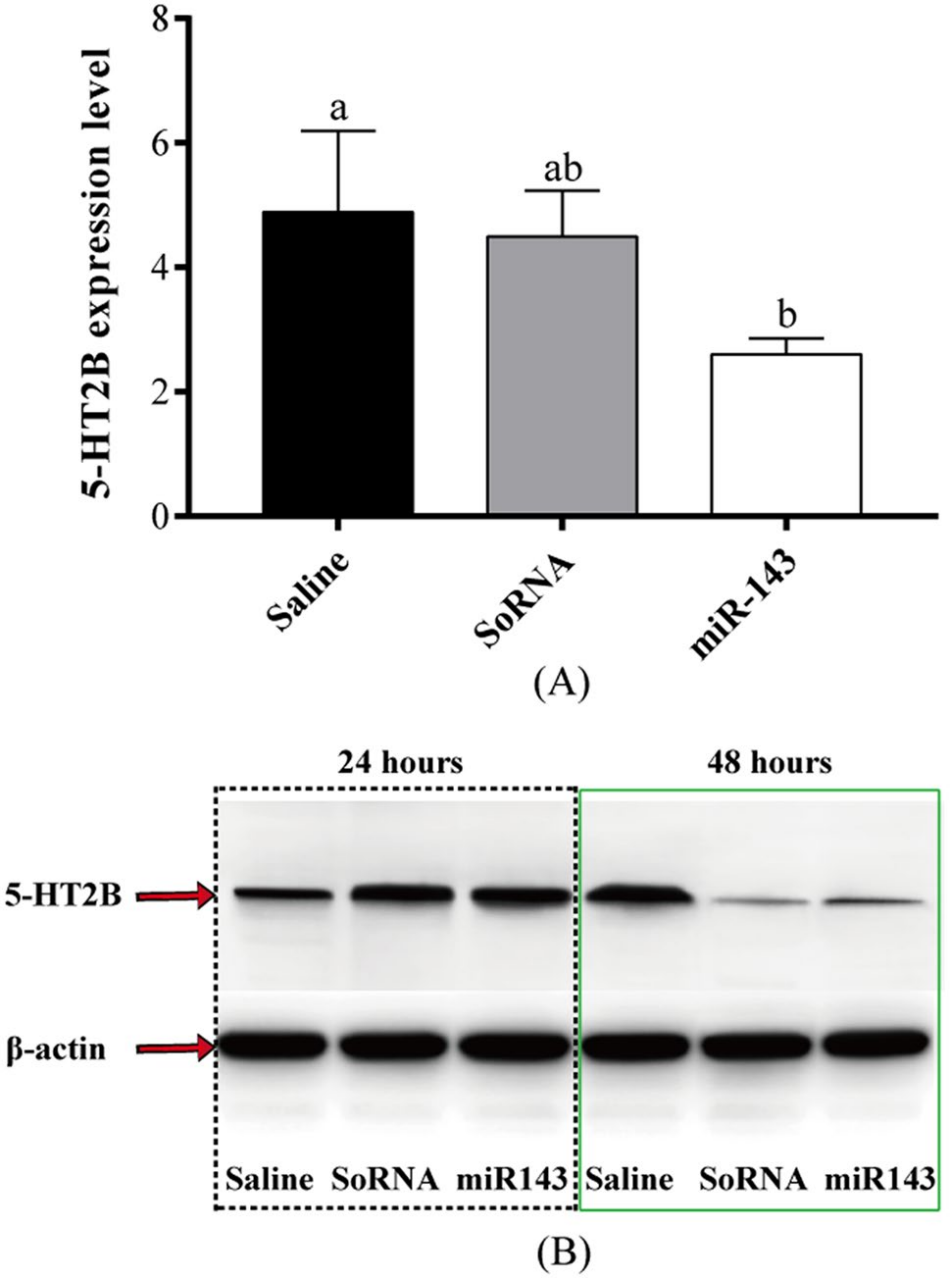
(**A**) 5-HT2B mRNA level after 48 h of miR-143 injection; (**B**) Relative expression levels of 5-HT2B protein 48 h after miR-143 injection as revealed using Western blot. The black dotted box indicates 5-HT2B protein level 24 h after injection and the green solid box indicates 5-HT2B protein level after 48 h. Different letters above the horizontal line indicate significant differences between the miR-143 injection and saline injection groups (*P* < 0.05).

After 48 h of miR-143 mimic injection, the 5-HT2B mRNA level is significantly decreased compared with those following saline or SoRNA injection (*P* < 0.05, Fig. 6A), indicated that miR-143 inhibits the expression of 5-HT2B mRNA in *E. sinensis*. Western blot analysis was used to verify the relative expression of 5-HT2B protein after miR-143 injection. The results demonstrated that there is no significant difference in 5-HT2B protein expression among the three groups after 24 h of injection. However, protein expression is significantly inhibited 48 h after miR-143 mimic injection (Fig. 6B). The above results indicated that miR-143 inhibits 5-HT2B expression in *E. sinensis*.

### Agonistic behavior observation after RNAi

Having established that miR-143 significantly inhibits the expression of 5-HT2B, we then investigated the effect of miR-143 on agonistic behavior in *E. sinensis* through interference with 5-HT2B expression. Agonistic behavior was observed 48 h after miR-143 injection. The results showed that miR-143 injection decreases contact number as compared with saline injection (Table 1), but does not influence approach number, fight number, or fight duration (Table 1). These data demonstrated that miR-143, which inhibits 5-HT2B expression, has no effect on agonistic behavior in *E. sinensis*.

**Table 1:** The numbers of approach, contact and fight in the groups of crustacean saline (saline), SoRNA, miR-143 injection. The different lowercase letters indicate a significant difference between the saline other injection groups (*P* < 0.05).

Pairs	*N*	Approach numbers	Contact numbers	Fight numbers	Fight duration (min)
Saline	4	8.25 ± 1.48	11.0 ± 4.58^a^	22.5 ± 6.34	23.12 ± 5.91
SoRNA	5	8.8 ± 3.06	3.8 ± 1.47^b^	15.6 ± 5.75	25.37 ± 6.73
miR-143	5	8.0 ± 1.67	4.0 ± 1.10^b^	23.8 ± 11.30	27.71 ± 10.44

The different lowercase letters indicate that there is a significant difference in the numbers of contact between saline and other injection groups (*P* < 0.05).

## Discussion

In the pond culture of *E. sinensis*, agonistic behavior directly impacted crab integrity, survival and growth and results in economic losses. Our final goal was to artificially regulate the agonistic behavior of crabs to reduce losses and improve commercial value. Therefore, we urgently needed to understand the mechanism of agonistic behavior in *E. sinensis* in order to develop new inhibitors or other methods to regulate agonistic behavior. According to our previous researches, we found that 5-HT can regulate the agonistic behavior through its downstream receptor (5-HT2) in *E. sinensis*^14^. And miRNAs can be used as inhibitors to regulate a variety of physiological pathways. Combined with these purposes, we explored that miR-143 could inhibit 5-HT2B expression in regulation the agonistic behavior in *E. sinensis*.

At first, we cloned and obtained the full-length sequence of the 5-HT2B gene in *E. sinensis*, which provided the basis for further study of its function. Through in vitro culture and luciferase reporter assays, the interaction between miR-143 and 5-HT2B 3′UTR was confirmed. In vivo injection experiments further demonstrated that miR-143 inhibits the expression of 5-HT2B mRNA and protein. Thus, miR-143 as a selective 5-HT2B inhibitor may be used to explore the function of the 5-HT2B gene in crustaceans. This is particularly important as the majority of previous researches on miR-143 was focused on its function in vertebrates, especially mammals.

According to these reports, miR-143 is significantly enriched in neural systems^23^. miR-143 not only participates in neuronal differentiation and development^24,25^, it also mediates the schizophrenia-related locomotor hyperactivity through the dopamine 2 receptor (DA2) in vertebrates^23^. DA2 plays a key role in the dopaminergic system and has been reported to mediate animal behavior. For example, manipulating the DA2 receptor with an agonist has been shown to reduce aggression in the cichlid fish (*Astatotilapia burtoni*)^26^. In addition, injection of a DA2 antagonist or iRNA knockdown increases locomotion in the migratory locust (*Locusta migratoria*)^27^. Our previous study also demonstrated that injection of a DA2 agonist decreases agonistic behavior in *E. sinensis*^14^, while Yin et al. (2019) reported that blocking miR-143 function is sufficient to cause pain-related behavior in intact mice.

The above studies in paragraph 2 demonstrated that miR-143 plays an important role in the regulation of animal behavior. However, there is a lack of research into the effect of miRNA on behavior in invertebrates. Accordingly, in this study, we have investigated the role of miR-143 in the regulation of crustacean behavior from a new perspective. This assumption was mainly based on the results of our previous research and database comparisons.

MiR-143 targets the 5-HT2B 3′UTR, as predicted using the miRanda database. Furthermore, the interaction between miR-143 and 5-HT2B was confirmed through in vitro culture experiments. We inferred that miR-143 may be involved in regulating animal behavior from the studies cited previously in this paper^20,23^. In fact, 5-HT as a neurotransmitter mediates animal behavior by acting upon the nervous system^28^. The 5-HT2B receptor belongs to a superfamily of G protein-coupled receptors and plays an important role in the downstream response to 5-HT^14,28^. 5-HT regulates agonistic behavior in crustaceans, as has been reported by many researchers^7,13,29^. However, there remains a lack of research into the function of the 5-HT2B receptor in regulating the behavior of crustaceans.

According to our previous study, the inhibition of the 5-HT2 receptor, which has two subtype receptors (5-HT2A and 5-HT2B) significantly decreases agonistic behavior in *E. sinensis*, but the expression of 5-HT2B mRNA does not change significantly after injection of the inhibitor^14^. Therefore, we further researched the function of 5-HT2B in regulating agonistic behavior in *E. sinensis*. Previously, Majeed et al*.* (2014) demonstrated that dysfunction of the 5-HT2B receptor during development negatively influences the locomotive behavior in *D. melanogaster*^15^. In another recent study, knockdown of the 5-HT2B gene decreases the sleep time in drosophila^30^. Furthermore, the level of 5-HT2B gene mRNA expression in *E. sinensis* is significantly different after fighting^9^. Therefore, the 5-HT2B gene was confirmed to be involved in mediating invertebrate behavior.

The downstream signaling pathways of 5-HT2B include cAMP/cGMP signaling^31,32^. According to a study on *P. clarkii*, cAMP has a modulatory effect on the rhythmic beating activity of the swimmeret motor neurons in crayfish^33^. Furthermore, Momohara et al. (2016) reported that injection of a cAMP mimic reduces crayfish aggression, indicating that the cAMP-PKA signaling pathway is essential for mediating loser and winner effects in agonistic behavior^34^. Moreover, cGMP signaling has been closely linked to agonistic behavior in crickets^35^, and the concentration of cGMP has been implicated in aggressive behavior in mice^36^. These findings suggest that 5-HT2B can affect several behaviors in invertebrates through its downstream signaling pathways.

However, our results shown that miR-143 inhibits 5-HT2B expression but does not influence agonistic behavior in *E. sinensis*. Nevertheless, our previous research has demonstrated that ketanserin does reduce agonistic behavior in *E. sinensis*^14^. Momohara et al. (2016) also proved that injection of 5HT2B receptor antagonist (methiothepin mesylate salt) does not affect the winner and loser effects in crayfish^34^. According to reports, ketanserin inhibits the activity of 5-HT2A rather than 5-HT2B^37,38^. Not only that, 5-HT2A has been confirmed that it can regulate the aggressive behavior in vertebrates, such as zebrafish, mice, and rats^39–41^. The above results indicate that 5-HT2A rather than 5-HT2B may be involved in agonistic behavior in *E. sinensis*.

Certainly, we will continue to probe the function of 5-HT2A and 5-HT2B in agonistic behavior using feasible methods in future studies. It should be noted, though, that the current study is the first to demonstrate the interaction between miR-143 and 5-HT2B 3′UTR. This finding may provide numerous possibilities for future research.

## Materials and methods

### Animal culture

Chinese mitten crabs were collected from Chongming Island (121° 30′–121° 40′ E, 31° 34′–31° 37′ N). And only intact and healthy males were used in this study. Intact intermolt crabs were maintained in separate opaque tanks (29.0 × 18.0 × 19.5 cm) for at least 7 days under single rearing conditions before the behavioral experiments. The tanks were filled to a depth of 12 cm with thoroughly aerated freshwater using a closed circulating water system (recirculating the same tank water). During the study, the crabs were kept at 20–24 °C, pH 7.6–7.8, and a dissolved oxygen concentration of at least 6.0 mg/L. Intact crabs were reared individually to avoid social contact. In addition, some of the crabs were maintained under group maintenance conditions and used for the in vitro culture and total RNA extraction experiments. A basal diet was used to feed the crabs once daily between 4:00 and 5:00 pm. The basal diet was a pellet feed designed for *E. sinensis* aquaculture and was purchased from ALPHA FEED (Shenzhen, China). The animal rearing methods were those used in our previous studies^9,14^. Due to the long experimental period, we irregularly purchased the crabs from July to December in 2019 for sampling. All animals were handled in accordance with guidelines established by the Animal Experiments Ethics Committee of Shanghai Ocean University for the care and use of laboratory animals.

### Cloning of full-length 5-HT2B cDNA

The core amino acid sequence of *E. sinensis* 5-HT2B was obtained from our transcriptome database and compared it with those of other crustaceans using BLASTX^22^. It was found to be highly homologous with those of *Cancer borealis* (KU710380.1) and *Panulirus interruptus* (AY550910.1) serotonin receptor type 2B mRNA. The total RNA of the cerebral ganglion and thoracic ganglia were extracted and mixed using RNAiso Plus reagent^42^, and reverse transcribed into 3′- and 5′-end cDNA templates using the SMARTer RACE 5′/3′ Kit (Clontech, USA) according to the manufacturer's instructions. We designed the gene-specific primers 5-HT2B 3′Outer, 5-HT2B 3′Inner, 5-HT2B 5′Outer, and 5-HT2B 5′Inner according to the core amino acid sequence using Primer Premier 5 (Table 2). The 3′- and 5′-ends of the 5-HT2B cDNA were cloned by rapid amplification of cDNA ends (RACE). Touchdown PCR and nested PCR were used to obtain specific products.

**Table 2:** Primers were used in cloning and characterizing the 5-HT2B gene.

Primer name	Primer sequence (5′–3′)
5-HT2B 3′Outer	TTCATCGCCAACGTCCTCATCTCG
5-HT2B 3′Inner	TTCAACAAGACCTTCAGACAAACG
5-HT2B 5′Outer	GATGACCAGCGGAATGTAGAAGCA
5-HT2B 5′Inner	GGCGTTCTGGGGAGTGGTCCAAAGA
5-HT2B-F	ATGCCCACCCTAGGGGACCTCAC
5-HT2B-R	TCAGAGGTGGTACTTCCTTGTGGTCTTGATGTC
Nest universal primer	CTAATACGACTCACTATAGGGC

For touchdown PCR, using a 25 µL mixture containing approximately 1.25 μL 3′- or 5′-end cDNA template, 2.5 μL 10 × UPM, 0.5 μL 5-HT2B 3′ or 5′Outer primer, 12.5 μL 2 × SeqAmp Buffer, 0.5 μL DNA polymerase, and 7.75 μL PCR-grade water to react. The mixture was treated as follows: 94 ℃ for 5 min; 94 ℃ for 30 s, 72 ℃ for 3 min, 5 cycles; 94 ℃ for 30 s, 70 ℃ for 30 s, 72 ℃ for 3 min, 5 cycles; 94 ℃ for 30 s, 68 ℃ for 30 s, 72 ℃ for 3 min, 30 cycles; 72 ℃ for 10 min, 16 ℃ for the cooling hold. Subsequently, the obtained reaction solution as a new cDNA template was used for nested PCR.

For nested PCR, 1 μL cDNA template (reaction solution obtained above diluted 2 to 5 times), 1 μL 5-HT2B 3′ or 5′Inner primer, 1 μL Nest Universal Primer (Table 2), and 10 μL Taq enzyme were mixed. The reaction conditions used were as follows: 94.

℃ for 5 min; 94 ℃ for 30 s, 60 ℃ for 30 s, 72 ℃ for 3 min, 30 cycles; 72 ℃ for 10 min, 16 ℃ for the cooling hold. The final amplification product was subjected to denaturing formaldehyde agarose gel electrophoresis and purified with a TIANgel Midi Purification Kit (TIANGEN, China)^42^. The DNA fragments was cloned into a pMD19-T vector (TaKaRa, Japan). Then, the vector was transformed into DH5α chemically competent cells (Sangon Biotech, Shanghai). The chemically competent cells were coated onto a plate containing ampicillin. The positive clones that contained the inserts of the expected size were isolated using denaturing formaldehyde agarose gel electrophoresis. Finally, the positive clones were sequenced by Sangon Biotech to confirm the correctness of the expected sequence.

The generated sequences were verified for similarity using the BLAST program (http://blast.ncbi.nlm.nih.gov/). Subsequently, the full length 5-HT2B and the 3′- and 5′-end sequences were obtained by sequencing and splicing. The open reading frame (ORF) finder website (NCBI) was used to identify the ORF, which was translated into an amino acid sequence. In order to verify the ORF sequence and eliminate individual uncertain bases, we designed forward (5-HT2B-F) and reverse (5-HT2B-R) specific primers for the beginning and end of the ORF to be used in PCR (Table 2).

### 5-HT2B sequence analysis

The 5-HT2B encoded protein was used to obtain the molecular mass and theoretical isoelectric point using the Compute pI/Mw tool (http://cn.expasy.org/tools/pi_tool.ht ml). The transmembrane domains of the protein sequence, the protein phosphorylation sites, and the N-glycosylation sites were predicted using TMHMM (http://www.cbs.dtu.dk/services/TMHMM), DISPHOS 1.3 (http://www.dabi.temple. edu/disphos/), and NetNGly 1.0 (http://www.cbs.dtu.dk/services/NetNGlyc/), respectively^42^. Finally, amino acid multiple sequence alignment and phylogenetic tree analysis were performed using MEGA 7.0 software.

### Prediction and identification of the miRNA target 5-HT2B 3′UTR

The two computational algorithms miRanda (http://www.microrna.org/) and TargetScan 5.1 (http://www.targetscan.org) were used to reveal that 5-HT2B 3′UTR is targeted by miRNAs. Then, the predicted miRNAs were compared with our previous mature miRNA transcriptome database^22^. As a result, miR-143, miR-200b, and miR-429 were predicted to target 5-HT2B 3′UTR. In order to further research the effectiveness of these three predicted miRNAs, their chemically synthesized analogues were used in this study (Sangon Biotech, Shanghai) (Table 3).

**Table 3:** The sequence of miRNAs which were used to chemical synthesis.

miRNA mimics name	miRNA sequence(5′–3′)
miR-143 mimics	GUCUGAGAUGAAGCACUGUAGCUC
miR-200b mimics	UAAUACUGCCUGGUAAUGAUGACG
miR-429 mimics	UAAUACUGUCUGGUAAAACCGU
SoRNA (NC)	UUGUACUACACAAAAGUACUG

### In vitro culture experiments

Six healthy crabs were cleaned with alcohol-soaked cotton balls and then sprayed with 75% alcohol before vivisection according to our previously published method^14^. For the first in vitro culture, we removed the hepatopancreas and rinsed it at least five times with sterile crustacean saline supplemented with 100 U/mL penicillin and 100 µg/mL streptomycin^43^. Then, the hepatopancreases were transferred to a 24-well aseptic culture plate and incubated for 16 h in 200 µL/well culture medium (Medium-199 containing 100 U/mL penicillin and 100 µg/mL streptomycin) at 27 ℃^44^.

Before tissue culture, miRNA mimics were dissolved in diethyl pyrocarbonate (DEPC)-treated water and then diluted with crustacean saline to a DEPC content of less than 5%, which was then added to the culture medium containing miR-143 mimic (0.04 μM), miR-200b mimic (0.04 μM), miR-429 mimic (0.04 μM), SoRNA (0.04 μM, negative control (NC), or saline (blank group). After the first culture, we found that only miR-143 significantly inhibited the expression of 5-HT2B mRNA in the hepatopancreas. To verify that miR-143 decreased the 5-HT2B mRNA level, we co-cultured the thoracic ganglion with the miR-143 mimic again in a second in vitro culture. Only three groups (miR-143, SoRNA, and saline) were used in this experiment, and the specific culture method was the same as above. After culturing, analysis of the expression levels of 5-HT2B to determine the effects of the miRNA. In the second in vitro culture, eighteen crabs were used to extract the thoracic ganglions which were divided equally into the three groups. All tissues were stored at − 80 ℃ until use.

### Plasmid construction

To further explore the interaction between miR-143 and its target gene 5-HT2B, miRanda (http://www.microrna.org/) was used to predict the potential target sequence of miR-143 to 5-HT2B 3′UTR. The psiCHECK2 plasmid was selected as the double luciferase vector. 5-HT2B 3′UTR (5′-CTCGAGAGGAGATGATCAACCTGGTCAC CTGGCTCGGCTACGCTTCGTCCATGGTCAACCCATTTTTCTACACGTTCTTCAACAAGACCTTCAGACAAACGTTCCTCAAGATCATGAAGTGCGACATCAAGACCACAAGGAAGTACCACCTCTGAGGCTTTAAGAATGGTTGTTATGTACTTTTATTCGTCCCCGCTACCCATCTTGTTATCCCTTTACCTGACTACCTCACCCCGCCCCGTTTCACCTGTTAGAACACCTCTATTCACCTGGCCCTCACCTGGTCACGCCGGCTCGCCCCTCCATCCCGCCCCCTAACCTCGCTATAGTGACTGCGAGCTGTTGAGGGCGGGAGAGAAGGGTGTTAAAGCGAGGTGTTGTTTAAAC-3′) was cloned into psiCHECK2 using the XhoI and PmeI restriction sites. The sequence of 5-HT2B 3′UTR complementary to the miR-143 seed sequence (TCGTCC CCGCTACCCATCTTG) was randomly mutated to TCATCCCATCTACCGTAGGC G, generating the wild-type psiCHECK2-5-HT2B-WT (named 5-HT2B-WT) and mutant psiCHECK2-5-HT2B-MUT (named as 5-HT2B-MUT) recombinant plasmids^45^. The templates for wild-type 5-HT2B 3′UTR and mutant 5-HT2B 3′UTR were derived from gene synthesis (GENERAL BIOSYSTEMS (Anhui) Co., Ltd.).

### Cell culture, transfection, and fluorescence assay

293T cells were cultured in DMEM containing 10% fetal bovine serum (Invitrogen Corporation, USA) at 37 ℃ and 5% CO_2_. The cells were divided into four groups and respectively co-cultured with 5-HT2B-WT + mimic NC, 5-HT2B-WT + miR-143 mimic, 5-HT2B-MUT + mimic NC, and 5-HT2B-MUT + miR-143 mimic using a cell transfection reagent (Invitrogen Corporation, USA) according to the manufacturer’s protocol. The miRNAs were synthesized by Sangon Biotech (Shanghai) according to the specific sequences (Table 3). After 48 h of co-culturing, the cell culture medium was removed and the reporter gene cell lysate was added (FENGHUISHENGWU, Hunan). After the cells were fully lysed, the luciferase activity was detected using a double luciferase detection kit (Promega Corporation, Madison, WI) using a multifunctional enzyme labeling instrument with a chemiluminescence detection function. The experiments were repeated three times.

### Verification of the miR-143 interference effect using RT-qPCR and Western blot

Based on the above in vitro experiments, we confirmed that miR-143 inhibits the expression of 5-HT2B. Subsequently, we further explored whether injection of the miR-143 mimic in vivo decreases 5-HT2B expression. In this experiment, the eighteen crabs were divided into a control group (injection of 20 µL crustacean saline), a negative control group (injection of SoRNA), and miR-143 mimic treatment group (injection of miR-143 mimic). In the SoRNA and miR-143 groups, each crab was injected in the third pereiopod with 20 µL of crustacean saline containing the SoRNA (0.04 μM) or miR-143 mimic (0.04 μM), respectively^46^. At 24 and 48 h after injection, the thoracic ganglia were extracted and the expression of 5-HT2B determined.

The total RNA of the thoracic ganglia was extracted using the RNAiso Plus reagent^42^. The mRNA levels for the 5-HT2B receptor were determined using an ABI 7500 Real-Time PCR System (Life Technology, USA). The 5-HT2B receptor was detected with gene-specific primers (Table 4), and 18S rRNA was used as the reference gene. The receptor expression levels were calculated using the 2^−ΔΔCt^ method^47^.

**Table 4:** Primers were selected for evaluating 5-HT2B expression level with RT-qPCR.

Primer name	Primer sequence (5′–3′)
18S-F	TCCAGTTCGCAGCTTCTTCTT
18S-R	AACATCTAAGGGCATCACAGA
*RT*-5-HT2B-F	AGGCGACGAAGGTTCTGGGTGTGGT
*RT*-5-HT2B-R	ACCAGGTTGATCATCTCCTCCCCGA

Before Western blot analysis, we designed a polypeptide sequence (TQPVANPTNSSEVQLC) as an antigen to immunize rabbits according to the 5-HT2B amino acid sequence, and a 5-HT2B-specific polyclonal antibody was prepared by Hangzhou Huaan Biotechnology Co., Ltd. Then, the total protein of the thoracic ganglia was extracted using a total protein extraction kit (Boster Biological Technology Co., Ltd.). Protein concentrations were determined using a bicinchoninic acid (BCA) assay kit (Sangon). We added an equal volume of protein buffer to the extracted protein and boiled the mixture for 5 min^48^. Then, the denatured protein solution (20 uL) was transferred to a comb hole and separated using 12% sodium dodecyl sulfate polyacrylamide gel electrophoresis (SDS-PAGE) at 70 V for 30 min then at 90 V until the indicator was within ~ 0.5 cm of the bottom of the gel. Before the end of electrophoresis, a PVDF membrane was immersed in methanol for 15 s then rinsed with deionized water for 2 min and immersed in transfer buffer for 5 min. The separated protein was then transferred onto the PVDF membrane at 200 mA for 70 min using an electro-transfer instrument that was kept in ice water. The PVDF membrane with the attached protein was sealed in 5% bovine serum albumin (BSA) for 2 h and placed in an incubation bag containing the primary antibodies (rabbit anti-5-HT2B, 1:1000) at 4 ℃ overnight. Subsequently, the PVDF membrane was incubated with goat anti-rabbit antibody (1:2000) and labeled with horseradish peroxidase (HRP) at room temperature for 2 h. After washing in 1X Tris-buffered saline, 0.1% TBST, the PVDF membrane was sensitized, developed, and fixed with X film in a darkroom. Rabbit β-actin was used as an internal reference in this experiment.

### Overexpression of miR-143 and behavioral observation

According to the results of RNAi, the crabs also were divided into a saline group, a SoRNA group (injection of 0.04 μM), and an miR-143 group (injection of 0.04 μM). Before injection, the intact crabs were fed for at least 7 days under single rearing conditions. After 48 h, the RNAi results showed that injection of the miR-143 mimic inhibits 5-HT2B expression. Thus, after miR-143 mimic injection, the crabs were raised under single rearing conditions for 48 h. Then, two crabs with a weight difference in the range 1%-4% were paired in a fresh tank (20.0 × 15.5 × 19.5 cm). The crabs with body weight in the range of 15-30 g were used in this study, and all pairs were randomly assigned to three groups. The crabs were injected with the same dose of a miRNA mimic. The tank contained water with a depth of 10 cm and was divided into equal halves by an opaque partition. One crab was placed on each side of the partition. After 10 min, the partition board was removed and the agonistic behavior of the crabs was observed using a high-definition camera (H.264 DVR) for 1 h. We recorded the number of approaches, contacts, and fight incidents and calculated the cumulative fighting time for each pairing. At least 6 pairs were observed in each group. This methodology is the same as that used in our previous study^9^.

### Statistical analysis

All data are expressed as mean ± SD. One-way analysis of variance was used for multiple group comparisons along with post-hoc LSD multiple range tests. Student’s t-test analysis was used for comparison between two groups. *P* < 0.05 and < 0.001 were taken as statistically significant differences.

### ARRIVE guidelines statement

This study was carried out in compliance with the ARRIVE guidelines (http://www.nc3rs.org.uk/page.asp?id=1357).

### Approval statement

All experimental protocols were approved by the Key Laboratory of Freshwater Aquatic Genetic Resources, Ministry of Agriculture, Shanghai Ocean University in this paper.

## Supplementary Information

Supplementary Information.

## References

[1] Davis KM, Huber R (2007). Activity patterns, behavioural repertoires, and agonistic interactions of crayfish: a non-manipulative field study. Behaviour.

[2] Peeke HV, Blank GS, Figler MH, Chang ES (2000). Effects of exogenous serotonin on a motor behavior and shelter competition in juvenile lobsters (*Homarus americanus*). J. Comp. Physiol. A.

[3] Tierney AJ, Greenlaw MA, Dams-O'Connor K, Aig SD, Perna AM (2004). Behavioral effects of serotonin and serotonin agonists in two crayfish species, *Procambarus clarkii* and *Orconectes rusticus*. Comp. Biochem. Physiol. Part A.

[4] Tierney AJ, Mangiamele LA (2001). Effects of serotonin and serotonin analogs on posture and agonistic behavior in crayfish. J. Comp. Physiol. A.

[5] Zhao Q, Pan LQ, Ren Q, Wang L, Miao JJ (2016). Effect of salinity on regulation mechanism of neuroendocrine-immunoregulatory network in *Litopenaeus vannamei*. Fish Shellfish Immunol..

[6] Momohara Y, Kanai A, Nagayama T (2013). Aminergic control of social status in crayfish agonistic encounters. PLoS ONE.

[7] Kravitz EA (1988). Hormonal control of behavior: amines and the biasing of behavioral output in lobsters. Science.

[8] Huber R, Smith K, Delago A, Isaksson K, Kravitz EA (1997). Serotonin and aggressive motivation in crustaceans: altering the decision to retreat. Proc. Natl. Acad. Sci. U. S. A..

[9] Yang XZ (2019). The serotonin or dopamine by cyclic adenosine monophosphate-protein kinase A pathway involved in the agonistic behaviour of Chinese mitten crab, *Eriocheir sinensis*. Physiol. Behav..

[10] Alekseyenko OV (2019). Serotonergic modulation of aggression in *Drosophila* involves GABAergic and cholinergic opposing pathways. CurrBiol.

[11] Aquiloni L (2012). Crustacean hyperglycemic hormone (CHH) as a modulator of aggression in crustacean decapods. PLoS ONE.

[12] Xu L, Pan L, Zhang X, Wei C (2019). Crustacean hyperglycemic hormone (CHH) affects hemocyte intracellular signaling pathways to regulate exocytosis and immune response in white shrimp *Litopenaeus vannamei*. Peptides.

[13] Johnson O, Becnel J, Nichols CD (2009). Serotonin 5-HT2 and 5-HT1A-like receptors differently modulate aggressive behaviors in *Drosophila melanogaster*. Neuroscience.

[14] Pang YY (2019). 5-HT2B, 5-HT7, and DA2 receptors mediate the effects of 5-HT and DA on agonistic behavior of the Chinese mitten crab (*Eriocheir sinensis*). ACS Chem. Neurosci..

[15] Majeed ZR, Stacy A, Cooper RL (2014). Pharmacological and genetic identification of serotonin receptor subtypes on *Drosophila* larval heart and aorta. J. Comp. Physiol. B Biochem. Syst. Environ. Physiol..

[16] Bartel DP (2004). MicroRNAs: genomics, biogenesis, mechanism, and function. Cell.

[17] Wang L (2018). Integrated microRNA and mRNA analysis in the pathogenic filamentous fungus *Trichophyton rubrum*. BMC Genom..

[18] Li XK, Jin P (2010). Roles of small regulatory RNAs in determining neuronal identity. Nat. Rev. Neurosci..

[19] Lane BJ, Kick DR, Wilson DK, Nair SS, Schulz DJ (2018). Dopamine maintains network synchrony via direct modulation of gap junctions in the crustacean cardiac ganglion. Elife.

[20] Dulcis D (2017). Neurotransmitter switching regulated by miRNAs controls changes in social preference. Neuron.

[21] Jensen KP (2009). A common polymorphism in serotonin receptor 1B mRNA moderates regulation by miR-96 and associates with aggressive human behaviors. Mol. Psychiatry.

[22] Pang Y (2020). Identification and integrated analysis of microRNA and mRNA expression profiles during agonistic behavior in Chinese mitten crab (*Eriocheir sinensis*) using a deep sequencing approach. Front. Genet..

[23] Wang P (2019). D2 receptor-mediated miRNA-143 expression is associated with the effects of antipsychotic drugs on phencyclidine-induced schizophrenia-related locomotor hyperactivity and with Neuregulin-1 expression in mice. Neuropharmacology.

[24] Olsen LC, O'Reilly KC, Liabakk NB, Witter MP, Saetrom P (2017). MicroRNAs contribute to postnatal development of laminar differences and neuronal subtypes in the rat medial entorhinal cortex. Brain Struct. Funct..

[25] Quaranta MT (2016). Identification of beta-Dystrobrevin as a direct target of miR-143: involvement in early stages of neural differentiation. PLoS ONE.

[26] Weitekamp CA, Nguyen J, Hofmann HA (2017). Social context affects behavior, preoptic area gene expression, and response to D2 receptor manipulation during territorial defense in a cichlid fish. Genes Brain Behav..

[27] Guo XJ, Ma ZY, Kang L (2015). Two dopamine receptors play different roles in phase change of the migratory locust. Front. Behav. Neurosci..

[28] Shiratori C (2017). Cyclic AMP-regulated opposing and parallel effects of serotonin and dopamine on phototaxis in the Marmorkrebs (marbled crayfish). Eur. J. Neurosci..

[29] Harlioglu MM, Harlioglu AG, Yonar SM, Duran TC (2014). Effects of dietary l-tryptophan on the agonistic behavior, growth, and survival of freshwater crayfish *Astacus leptodactylus eschscholtz*. Aquacult. Int..

[30] Qian YJ (2017). Sleep homeostasis regulated by 5HT2b receptor in a small subset of neurons in the dorsal fan-shaped body of drosophila. Elife.

[31] Amireault P, Dube F (2005). Intracellular cAMP and calcium signaling by serotonin in mouse cumulus-oocyte complexes. Mol. Pharmacol..

[32] Nebigil CG (2001). Developmentally regulated serotonin 5-HT2B receptors. Int. J. Dev. Neurosci..

[33] Mita A, Yoshida M, Nagayama T (2014). Nitric oxide modulates a swimmeret beating rhythm in the crayfish. J. Exp. Biol..

[34] Momohara Y, Minami H, Kanai A, Nagayama T (2016). Role of cAMP signalling in winner and loser effects in crayfish agonistic encounters. Eur. J. Neurosci..

[35] Aonuma H (2007). NO/cGMP system and biogenic amine system in agonistic behavior in the cricket. Comp. Biochem. Physiol. A Mol. Integr. Physiol..

[36] Hotchkiss AK (2005). Aggressive behavior increases after termination of chronic sildenafil treatment in mice. Physiol. Behav..

[37] Monte AP (1996). Dihydrobenzofuran analogues of hallucinogens. 3. Models of 4-substituted (2,5-dimethoxyphenyl)alkylamine derivatives with rigidified methoxygroups. J. Med. Chem..

[38] Xu W-J (2020). Involvement of 5-HT2A, 5-HT2B and 5-HT2C receptors in mediating the ventrolateral orbital cortex-induced antiallodynia in a rat model of neuropathic pain. NeuroReport.

[39] Kanno H (2009). Effect of yokukansan, a traditional Japanese medicine, on social and aggressive behaviour of para-chloroamphetamine-injected rats. J. Pharm. Pharmacol..

[40] Iba H (2019). Effect of yokukansan and yokukansankachimpihange on aggressive behavior, 5-HT receptors and arginine vasopressin expression in social isolation-reared mice. Biol. Pharm. Bull..

[41] Mueller TE (2020). Role of the serotonergic system in ethanol-induced aggression and anxiety: a pharmacological approach using the zebrafish model. Eur. Neuropsychopharmacol..

[42] Yang X (2018). Cloning and functional characterization of the DA2 receptor gene in Chinese mitten crab (*Eriocheir sinensis*). PLoS ONE.

[43] Xu Y, Ye HH, Ma J, Huang HY, Wang GZ (2010). Primary culture and characteristic morphologies of neurons from the cerebral ganglion of the mud crab, *Scylla paramamosain*. Vitro Cell. Dev. Biol. Anim..

[44] Arief B, Seiichi W, Sulistiono S, DodyDharmawan T, Yushinta F (2007). Development of mud crab (*Scylla olivaceous herbst*) oocyte after in vitro culture with thoracic ganglion extracts of estuarine crabs (neoepisesarmalafondijacqunot and lucas). Biotropia.

[45] Huang Y, Han KK, Wang W, Ren Q (2017). Host microRNA-217 promotes white spot syndrome virus infection by targeting tube in the Chinese mitten crab (*Eriocheir sinensis*). Front. Cell. Infect. Microbiol..

[46] Yang XZ (2018). Thehyperglycemiceffect of melatonin in the Chinese mitten crab *Eriocheir sinensis*. Front. Physiol..

[47] Livak KJ, Schmittgen TD (2001). Analysis of relative gene expression data using real-time quantitative PCR and the 2(-Delta DeltaC(T)) method. Methods.

[48] Wu DM, Zhang YT, Lu J, Zheng YL (2018). Effects of microRNA-129 and its target gene c-Fos on proliferation and apoptosis of hippocampal neurons in rats with epilepsy via the MAPK signaling pathway. J. Cell. Physiol..

