# Supplementary figures and images for: Effects of miR-143 and its target receptor 5-HT2B on agonistic behavior in the Chinese mitten crab (Eriocheir sinensis)
Source: Sci Rep. 2021 Feb 24;11:4492. doi: 10.1038/s41598-021-83984-6 (PMC7904944; doi:10.1038/s41598-021-83984-6)

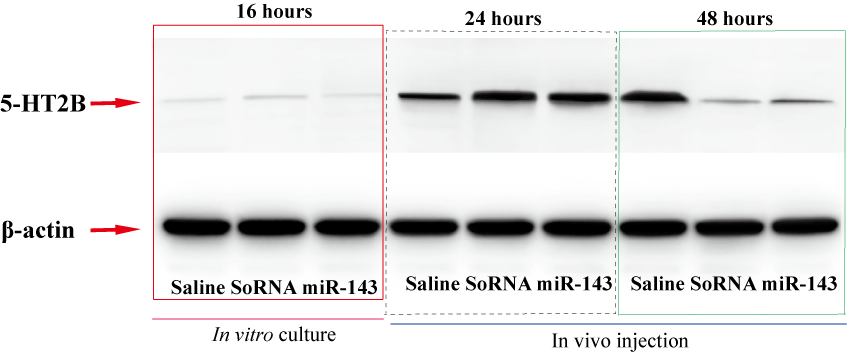

Supplement: Supplementary file 1 — Supplementary Information. [file 41598_2021_83984_MOESM1_ESM.tif]
